# Diagnostic accuracy of blood sucrose as a screening test for equine gastric ulcer syndrome (EGUS) in adult horses

**DOI:** 10.1186/s13028-017-0284-1

**Published:** 2017-03-11

**Authors:** Michael Hewetson, Ben William Sykes, Gayle Davina Hallowell, Riitta-Mari Tulamo

**Affiliations:** 1 Department of Equine and Small Animal Medicine, Faculty of Veterinary Medicine, University of Helsinki, Helsinki, Finland; 2 School of Veterinary Sciences, University of Queensland, Brisbane, Australia; 3 School of Veterinary Medicine and Science, University of Nottingham, Nottingham, UK; 4 Department of Companion Animal Clinical Studies, Faculty of Veterinary Science, University of Pretoria, Onderstepoort, South Africa

**Keywords:** Sucrose, Equine, Ulcer, Glandular, Squamous, EGUS, EGGD, ESGD, Permeability, Sensitivity, Specificity

## Abstract

**Background:**

Equine gastric ulcer syndrome (EGUS) is common in adult horses, particularly those involved in performance disciplines. Currently, detection of EGUS by gastroscopy is the only reliable ante mortem method for definitive diagnosis; however it is unsuitable as a screening test because it is expensive, time consuming, and is not readily available to most veterinarians. Sucrose permeability testing represents a simple, economical alternative to gastroscopy for screening purposes, and the feasibility of this approach in the horse has been previously reported. The aim of this study was to determine the diagnostic accuracy of blood sucrose as a screening test for EGUS in a large group of adult horses with and without naturally occurring gastric disease.

**Results:**

One hundred and one adult horses with or without naturally occurring gastric ulceration were studied. The diagnostic accuracy of blood sucrose for diagnosis of gastric lesions (GL), glandular lesions (GDL), squamous lesions (SQL), and clinically significant lesions (CSL) at 45 and 90 min after administration of 1 g/kg of sucrose via nasogastric intubation was assessed using receiver operator characteristics (ROC) curves and calculating the area under the curve (AUC). For each lesion type, sucrose concentration in blood was compared to gastroscopy, as the gold standard, and sensitivities (Se) and specificities (Sp) were calculated across a range of sucrose concentrations. Ulcer grading was performed blindly by one observer; and the results were validated by comparing them with that of two other observers, and calculating the level of agreement. Cut-off values were selected manually to optimize Se. The prevalence of GL, GDL, SQL, and CSL was 83, 70, 53 and 58% respectively. At the selected cut-offs, Se ranged from 51 to 79% and Sp ranged from 43 to 72%, depending upon the lesion type and time of sampling.

**Conclusions:**

Blood sucrose is neither a sensitive or specific test for detecting EGUS in this population of adult horses with naturally occurring gastric ulceration. Further studies aimed at evaluating the performance characteristics of the test in different study populations are warranted. Given the limitations of endoscopy, due consideration should also be given to alternative methods for comparison of blood sucrose with a gold standard.

**Electronic supplementary material:**

The online version of this article (doi:10.1186/s13028-017-0284-1) contains supplementary material, which is available to authorized users.

## Background

Equine gastric ulcer syndrome (EGUS) is a term used to describe erosive and ulcerative diseases of the equine stomach; and can be further classified into equine squamous gastric disease (ESGD) and equine glandular gastric disease (EGGD) based on the anatomical region affected [1]. EGUS is common in horses and although the clinical ramifications of this disease have as yet, not been completely elucidated, it remains an important disease in the equine industry. Performance horses are particularly susceptible, with 47–100% of Thoroughbred racehorses [2–5], 44–87% of Standardbred racehorses [6–8], 33–93% of endurance horses [9, 10] and 58–64% of show and sport horses [11, 12] found to have gastric lesions on gastroscopy. Non-performance horses are also susceptible to EGUS, with ulcers found in the gastric mucosa of 11–67% of sedentary horses and horses that partake in less strenuous activities [13–15].

Currently, detection of EGUS by gastroscopy is the only reliable ante mortem method for definitive diagnosis in horses [16] and is considered the gold standard against which all other diagnostic tests are compared [1]. Disadvantages of gastroscopy are that it’s not readily available to most veterinarians, it is an inefficient expenditure of time, and it requires a minimum level of expertise to perform and interpret. Furthermore, gastroscopy is costly to the client and with an increase in public awareness of EGUS and its popularity as a ‘catch-all’ diagnosis for poor performance in sport horses, many owners are electing to treat their horses on an empirical basis without the benefit of a definite diagnosis. Given the current economic climate and the rising costs of omeprazole, it is easy then to imagine that owners and veterinarians would be interested in using an economical screening test to rule out gastric ulcers. Such a screening test should ideally have a high sensitivity as it will correctly identify most horses with gastric ulcers, remembering that many horses with EGUS will not demonstrate clinical signs, and are considered to have ‘silent’ or non-clinical gastric ulceration [14, 17–20].

Sucrose permeability testing represents a simple, economical alternative to gastroscopy for screening purposes, and the feasibility of this approach in the horse has been previously reported [21–23]. Because of its large molecular size (342 Da), sucrose is not able to permeate across healthy gastrointestinal mucosa, but it has been reported to cross the mucosa in the presence of gastrointestinal disease, presumably due to an changes in intestinal tight junction permeability or directly through gaps in the epithelium caused by erosion or ulceration [24–26]. The efficiency of the mucosal disaccharidases and the monosaccharide transport systems in the equine small intestine has been established by a series of oral disaccharide and monosaccharide tolerance tests, and it has been demonstrated that adult horses are fully capable of rapidly hydrolyzing sucrose [27, 28]. Furthermore, sucrase has the highest activity in the duodenum of the horse, with concentrations similar to those reported in the intestine of other non-ruminant species [29]. If present in blood, sucrose is cleared via the urine; it is not metabolized and the body does not produce it [30, 31]. Therefore, increased amounts of sucrose in blood after an oral dose is site specific for increased gastric permeability, and can be used to predict the presence of gastric disease [32–38].

The objective of this study was to determine the diagnostic accuracy of blood sucrose as a potential screening test for EGUS in adult horses by comparing it to gastroscopy as the gold standard.

## Methods

### Study design

The study was conducted as a blind comparison to a gold standard.

### Study population

One hundred and one adult horses were eligible for inclusion in the study and were recruited from horses that had been referred to the University of Helsinki Equine Teaching Hospital, Finland for gastroscopy and from a local riding center. The horses were used for a wide range of equestrian activities, ranging from dressage to racing, and were recruited on the assumption that up to 53% of them would be affected by naturally occurring gastric ulceration of EGUS severity score ≥2 [14, 16]. Horses were excluded from the study if they had received non-steroidal anti-inflammatory drugs or omeprazole within 7 days prior to testing. This was done to avoid confounding changes in gastric permeability secondary to administration of these drugs [23, 39, 40].

### Gastroscopy

Owners were asked to withhold food from their horses for 16 h and water for 6 h prior to sucrose testing. Following completion of fasting, blood samples (10 ml) were collected in vacuumed clot tubes from the jugular vein; horses were sedated with a combination of intravenous detomidine hydrochloride (10 µg/kg body weight (BW)^1^ and butorphanol (0.025 mg/kg BW)^2^; and gastroscopy was performed using a previously described technique [21].

All endoscopic examinations were recorded and archived. For each horse, video recordings and still-frame images were taken of the stomach from the right side of the stomach along the margo plicatus, the dorsal part of the fundus, the greater curvature along the margo plicatus, the lesser curvature along the margo plicatus, the glandular mucosa in the region of the pylorus and the proximal duodenum [41].

### Administration of sucrose and collection of samples

Immediately following gastroscopy, 1 g/kg BW of sucrose^3^ was administered as a 10% solution via nasogastric tube to each horse. Blood samples (10 ml) were then collected in vacuumed clot tubes from the jugular vein at 45 and 90 min after administration of sucrose. These time points were chosen based upon data from a previous study which indicated that peak sucrose concentrations occur approximately between 45 and 90 min after sucrose administration [21]. Horses were not given access to food until the final blood sample had been collected to prevent ingestion of sucrose that may have been present in the food. Following blood collection, the serum was separated by centrifugation (10 min at 2000×*g*) and then stored in a freezer at −80 °C until analysis.

### Lesion assessment

Following completion of data collection, video recordings and still-frame images from each horse were reviewed independently by a board-certified internist (BS) who was blinded to the results of the sucrose assay. For each set of videos/images, the observer was asked to answer a set of dichotomous (yes or no) questions: does the horse have (1) gastric lesions? (2) glandular lesions? (3) squamous lesions? and (4) are the gastric lesions clinically significant? The term “gastric lesion” was used to describe lesions throughout the gastric mucosa and is synonymous with the term EGUS. In contrast, the terms “glandular lesion” and “squamous lesion” were used to differentiate the two different anatomical regions of the equine stomach and are synonymous with the term EGGD and ESGD respectively [1]. Clinically significant gastric lesions were used as a proxy indicator of ulcer severity and were defined as lesions that the observer would consider severe enough to warrant treatment. The term ‘lesion’ rather than ‘ulceration’ was used to enable the observer to report on the presence of other types of lesions (e.g. erosions) in addition to ulceration, as any damage to the mucosa of the stomach has the theoretical potential to increase permeability to sucrose [24–26].

### Inter-observer agreement

In order to assess the validity of the gastroscopy assessment, the observations for each horse were compared with observations made by two other board certified internists on the same set of video recordings and still-frame images (GH, MH), and the level of agreement was calculated.

### Sample processing and analyses

Serum was analyzed for sucrose using a previously validated gas chromatography-flame ionization detection (GC-FID) assay for quantifying sucrose in equine serum [42].

### Statistical analysis

The overall diagnostic accuracy of blood sucrose for diagnosis of GL, GDL, SQL and CSL was assessed using receiver operator characteristics (ROC) curves and calculating the area under the curve (AUC). For each diagnostic criterion, sucrose concentration in blood at 45 and 90 min was compared with gastroscopy as the gold standard; and sensitivities (Se), specificities (Sp), positive predictive values (PPV) and negative predictive values (NPV) were calculated across a range of sucrose concentrations. Optimal cut-off values were then selected manually to optimize sensitivity and provide a practical threshold for practitioners in the field when screening horses for EGUS. Confidence intervals were set at 95% (95% CI).

Inter-observer agreement was summarized as the percentage of perfect (100%) agreements between observers for each diagnostic criterion, and a kappa coefficient (K) was calculated.

Statistical analyses were performed with R for Windows^®^ version 3.0.2^4^.

## Results

### Horses

One hundred and one adult horses were accepted into the study; 59 mares, 4 stallions, and 38 geldings. Horses ranged from two to 22 years of age (median, 9.9 years). Body weight ranged from 400 to 683 kg (median, 518 kg). Breeds included 37/101 Warmbloods, 25/101 Finnhorses, 34/101 Standardbreds, 3/101 Welsh Ponies, 1/101 Trakhener, and 1/101 Arab. Horses were used for a variety of purposes, including eventing, show jumping, dressage, trotting and general riding purposes. Fifty-three horses were demonstrating clinical signs suggestive of EGUS at the time of gastroscopy.

### Gastroscopy

The overall prevalence of gastric lesions (ulcers or erosions) was 83%. Lesions were most common in the glandular mucosae (70%), followed by the squamous mucosae (53%). Fifty eight percent of the horses had gastric lesions that were severe enough to be considered clinically significant i.e. requiring treatment. Squamous lesions were most frequently observed in the region of the cardia and along the lesser curvature of the stomach adjacent to the margo plicatus; and consisted primarily of small single ulcers characteristic of EGUS severity score ≤2. Glandular lesions were exclusively observed around the pylorus and consisted primarily of focal raised hemorrhagic or fibrinous lesions.

### Sucrose permeability

All horses tolerated sucrose permeability testing and no adverse effects were noted following administration of the sucrose solution. On analysis of the serum samples, all horses demonstrated an increase in serum sucrose concentration over time, with peak serum sucrose concentrations occurring 90 min after administration of the sucrose solution.

The mean ± SD serum sucrose concentration at 45 min was 6.85 ± 4.90 µmol/l for normal horses (n = 17); 9.66 ± 9.16 µmol/l for horses with GL (n = 84); 9.44 ± 9.27 µmol/l for horses with GDL (n = 71); 10.56 ± 8.66 µmol/l for horses with SQL (n = 54); and 10.43 ± 9.22 µmol/l for horses with CSL (n = 59). The mean ± SD serum sucrose concentration at 90 min was 7.22 ± 4.65 µmol/l for normal horses (n = 17); 10.29 ± 8.12 µmol/l for horses with GL (n = 84); 9.86 ± 7.54 µmol/l for horses with GDL (n = 71); 11.53 ± 8.17 µmol/l for horses with SQL (n = 54); and 11.24 ± 8.55 µmol/l for horses with CSL (n = 59).

### Diagnostic accuracy of blood sucrose for diagnosis of EGUS

**Fig. 1:**
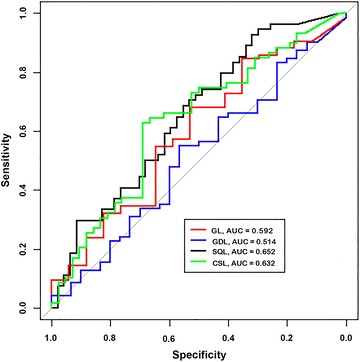
ROC curves for blood sucrose concentration when used to distinguish between normal horses and horses with GL, GDL, SQL and CSL at 45 min after administration of 1 g/kg of sucrose via nasogastric intubation. *AUC* area under the curve

**Fig. 2:**
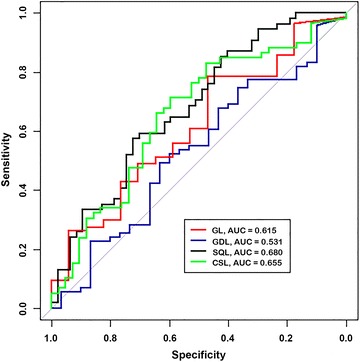
ROC curves for blood sucrose concentration when used to distinguish between normal horses and horses with GL, GDL, SQL and CSL at 90 min after administration of 1 g/kg of sucrose via nasogastric intubation. *AUC* area under the curve

ROC curves for each diagnostic criterion at 45 and 90 min after sucrose administration are illustrated in Figs. 1 and 2.

#### Gastric lesions

The AUC ± 95% CI for blood sucrose concentration when used to distinguish between normal horses and horses with GL at 45 and 90 min was 0.59 (0.44–0.74) and 0.62 (0.47–0.76) respectively. Sucrose concentrations of 4.61 µmol/l at 45 min and 4.57 µmol/l at 90 min were selected as the optimal cut-offs for discriminating between normal horses and horses with GL. The Se, Sp, PPV and NPV of blood sucrose at 45 and 90 min for diagnosis of GL using the selected cut-off values are depicted in Table 1.

**Table 1:** Sensitivity, specificity, positive and negative predictive values of blood sucrose for diagnosis of GL in horses after administration of 1 g/kg of sucrose via nasogastric intubation

Time of sampling (min)	Cut-off (µmol/l)	Disease present	Disease absent	Sp (%)	Se (%)	NPV (%)	PPV (%)
				95% CI	95% CI	95% CI	95% CI
45	4.61	84	17	52.9	67.9	25.0	87.7
				28–77	57–78	12–42	77–95
90	4.57	84	17	47.1	78.6	30.8	88.0
				23–72	68–87	14–52	78–94

*GL* gastric lesions, *Se* sensitivity, *Sp* specificity, *NPV* negative predictive value, *PPV* positive predictive value, *95% CI* 95% confidence intervals

#### Glandular lesions

The AUC ± 95% CI for blood sucrose concentration when used to distinguish between normal horses and horses with GDL at 45 and 90 min was 0.51 (0.39–0.64) and 0.53 (0.40–0.66) respectively. Sucrose concentrations of 5.80 µmol/l at 45 min and 6.05 µmol/l at 90 min were selected as the optimal cut-offs for discriminating between normal horses and horses with GDL. The Se, Sp, PPV and NPV of blood sucrose at 45 and 90 min for diagnosis of GDL using the selected cut-off values are depicted in Table 2.

**Table 2:** Sensitivity, specificity, positive and negative predictive values of blood sucrose for diagnosis of GDL in horses after administration of 1 g/kg of sucrose via nasogastric intubation

Time of sampling (min)	Cut-off (µmol/l)	Disease present	Disease absent	Sp (%)	Se (%)	NPV (%)	PPV (%)
				95% CI	95% CI	95% CI	95% CI
45	5.80	71	31	48.1	50.7	30.0	69.2
				30–67	39–63	18–45	55–81
90	6.05	71	31	43.3	66.2	35.1	73.4
				26–63	54–77	20–53	61–84

*GL* gastric lesions, *Se* sensitivity, *Sp* specificity, *NPV* negative predictive value, *PPV* positive predictive value, *95% CI* 95% confidence intervals

#### Squamous lesions

The AUC for blood sucrose concentration when used to distinguish between normal horses and horses with SQL at 45 and 90 min was 0.65 (0.55–0.76) and 0.68 (0.58–0.79) respectively. Sucrose concentrations of 7.86 µmol/l at 45 min and 8.24 µmol/l at 90 min were selected as the optimal cut-offs for discriminating between normal horses and horses with SQL. The Se, Sp, PPV and NPV of blood sucrose at 45 and 90 min for diagnosis of SQL using the selected cut-off values are depicted in Table 3.

**Table 3:** Sensitivity, specificity, positive and negative predictive values of blood sucrose for diagnosis of SQL in horses after administration of 1 g/kg of sucrose via nasogastric intubation

Time of sampling (min)	Cut-off (µmol/l)	Disease present	Disease absent	Sp (%)	Se (%)	NPV (%)	PPV (%)
				95% CI	95% CI	95% CI	95% CI
45	7.86	54	47	68.1	50.0	54.2	64.3
				53–81	36–64	41–67	48–79
90	8.24	54	47	72.3	57.4	59.7	70.5
				57–84	43–71	46–72	55–83

*SQL* squamous lesions, *Se* sensitivity, *Sp* specificity, *NPV* negative predictive value, *PPV* positive predictive value, *95% CI* 95% confidence intervals

#### Clinically significant gastric lesions

The AUC for blood sucrose concentration when used to distinguish between normal horses and horses with CSL at 45 and 90 min was 0.63 (0.52–0.74) and 0.66 (0.55–0.77) respectively. Sucrose concentrations of 4.61 µmol/l at 45 min and 5.87 µmol/l at 90 min were selected as the optimal cut-offs for discriminating between normal horses and horses with clinically significant lesions. The Se, Sp, PPV and NPV of blood sucrose at 45 and 90 min for diagnosis of CSL using the selected cut-off values are depicted in Table 4.

**Table 4:** Sensitivity, specificity, positive and negative predictive values of blood sucrose for diagnosis of CSL in horses after administration of 1 g/kg of sucrose via nasogastric intubation

Time of sampling (min)	Cut-off (µmol/l)	Disease present	Disease absent	Sp (%)	Se (%)	NPV (%)	PPV (%)
				95% CI	95% CI	95% CI	95% CI
45	4.61	59	42	50.0	74.6	58.3	67.7
				34–66	62–85	41–75	55–79
90	5.87	59	42	52.4	76.3	61.1	69.2
				36–68	63–86	44–77	57–80

*CSL* clinically significant lesions, *Se* sensitivity, *Sp* specificity, *NPV* negative predictive value, *PPV* positive predictive value, *95% CI* 95% confidence intervals

### Inter-observer agreement

When asked to answer if each horse has (1) gastric lesions; (2) glandular lesions; (3) squamous lesions; and (4) clinically significant gastric lesions, perfect agreement between-observers within the 101 sets of observations was achieved on average, in 83% (K = 0.50; *P* < 0.0001; 95% CI 0.24–0.76); 78% (K = 0.57; *P* < 0.0001; 95% CI 0.39–0.75); 74% (K = 0.65; *P* < 0.0001; 95% CI 0.53–0.77); and 75% (K = 0.62; *P* < 0.0001; 95% CI 0.48–0.75) of the cases respectively.

## Discussion

The objective of this study was to validate the sucrose blood test as a screening test for EGUS in adult horses by determining its performance characteristics in a large group of horses with and without naturally occurring gastric disease. ROC curve analysis was used to visually demonstrate the cut-off dependency of the test across a range of sucrose concentrations and to provide an estimate of the overall diagnostic accuracy of the test that is independent of specific cut-off values or prevalence of gastric lesions in the study population. For this study, ROC curves of true positive rates (Se) against false positive rates (1-Sp) were plotted using blood sucrose concentrations from normal horses, and horses with GL, GDL, SQL and CSL at 45 and 90 min after administration of sucrose (Figs. 1, 2). The AUC’s in each plot represents a summary of the overall diagnostic accuracy of the test by combining accuracy over a range of cut-offs, with a value approaching 1.0 indicating perfect discrimination and 0.5 representing zero discrimination. Using an arbitrary guideline, the AUC can be used to distinguish between a non-informative (AUC = 0.5); less accurate (0.5 < AUC ≤ 0.7); moderately accurate (0.7 < AUC ≤ 0.9); highly accurate (0.9 < AUC < 1); and perfect test (AUC = 1) [43]. Depending upon the lesion type and time of sampling, the AUC for the blood sucrose test ranged from 0.51 to 0.68, indicating that blood sucrose concentration is poor at discriminating between normal horses and horses with EGUS and is therefore not considered to be a very accurate test.

Because the AUC summarizes the ROC curve as a whole, and therefore attributes the same weighting to both relevant and irrelevant parts of the curve [44], cut-off values were inserted on the continuous scale of test results that allowed calculation of Se and Sp for horses with GL, GDL, SQL and CSL at each time point. Using the selected cut-offs, the Se and Sp of the blood sucrose test for detecting the presence or absence of EGUS was low (Tables 1, 2, 3, 4), confirming the poor diagnostic accuracy of the test in this study population.

It is not immediately evident why the sucrose blood test has a poor diagnostic accuracy in adult horses despite previous literature to suggest otherwise [21–23]. In this study, there was a predominance of glandular lesions (70%) whereas in previous studies, sucrose permeability was assessed primarily on horses with squamous lesions; and it may be that there are fundamental differences in the permeability of the sucrose molecule between the glandular and squamous epithelium. It has been found that gap junctional intercellular communication (GJIC) plays an important role in the gastric mucosal defense system, and loss of GJIC is associated with ulcer formation. A recent study demonstrated the presence of specific gap junctions in the glandular mucosa of the equine stomach, however these gap junctions were absent in the squamous mucosa of the stomach [45]. This suggests that there are significant differences in the permeation pathway of the glandular vs. the squamous epithelium which may explain (in part) why in this study population, with a predominace of glandular ulcers, the sucrose blood test had a poorer diagnostic accuracy than expected. Furthermore, glandular lesions are often smaller in cross-sectional area and are usually not ulcerative per se, but rather erosive or may even consist of intact mucosa with hyperemia [1]. In such cases, it is possible that sucrose is less likely to permeate in quantities large enough to appreciate differences between affected and unaffected horses, although this has yet to be substantiated.

The authors do recognize however, that the sensitivity and specificity for squamous lesions was also poor, albeit less so than for glandular ulcers. Another factor to consider therefore, is the fact that in this particular study, very few of the squamous lesions were extensive or demonstrated areas of apparent deep ulceration characteristic of EGUS severity score ≥3 [1]. It is therefore possible that in such cases, the total surface area for sucrose permeation was too small to differentiate between affected and unaffected horses. Based on this premise, re-analysis of the data using a scoring system that takes into account not only the severity of the lesion, but also the number of lesions should be considered [46].

Alternatively, the validity of the gold standard itself can be questioned. It may be that the sucrose test is too sensitive and may detect slight and clinically insignificant mucosal damage that cannot be seen on endoscopy, thus limiting its use in clinical decision-making regarding gastric ulceration [33]. We postulate that sucrose permeability is in fact an accurate representation of the true mucosal integrity of the stomach based on a number of previous publications documenting its effectiveness in both humans and other species [35, 36, 38, 47]; and that assessment via endoscopy is under- or overestimating the severity or depth of gastric lesions. This is based on the fact that assessment of lesion severity (and even the presence or absence of lesions) using gastroscopy is subjective, and agreement between observers for endoscopic diagnosis is notoriously poor, particularly if they are inexperienced [48, 49]. Furthermore, it has been demonstrated that there is a poor correlation between endoscopic assessment of gastric ulcers ante mortem and histological appearance at necropsy [46, 50]. Because of these limitations, an attempt was made in this study to determine if the gold standard was reproducible between-observers. All assessments made by the observer were compared with assessments made by two other board certified internists that have experience with gastroscopy, and the level of agreement for each outcome was determined. Agreement was moderate [51], but still unacceptably low, and it is possible that in the hands of different observers, the diagnostic accuracy of the test will vary. Considering these limitations, it is the authors’ opinion that histopathology rather than endoscopy should be utilized as a gold standard for comparison in future gastric permeability studies. Alternatively, Bayesian statistical approaches that are used for evaluation of diagnostic tests in the absence of a gold standard test should be considered [52].

The choice to include the severity of gastric ulceration in the study was based on the premise that the sucrose blood test would be able to differentiate between severe and less severe lesions, enabling practitioners to select cases for treatment based upon the outcome of the test. Unfortunately there are no grading systems that can be used interchangeably for horses with both ESGD and EGGD [53], and so the authors elected to use the concept of a ‘clinically significant gastric lesion’ as a proxy indicator of ulcer severity, where clinically significant lesions were defined as lesions that the observer would consider severe enough to warrant treatment if seen in a clinical case. While the authors recognize that this is not a perfect solution, as clinicians will usually use both gastroscopic appearance of lesions in combination with the clinical history to determine clinically significance, the authors believe that this proxy is the best possible compromise. While a scoring system (e.g. EGUS 0-4) would have been more objective, the fact that it cannot be used for EGGD makes it impossible to be used in this study. In future, assessment of both clinical and endoscopic outcomes when determining the diagnostic accuracy of the sucrose test is recommended.

When conducting a validation study to determine the diagnostic accuracy of a test, it is essential that the study include an appropriate spectrum of subjects which is representative of the population for which the test is intended. We aimed to determine the diagnostic accuracy of the sucrose blood test as a screening test for EGUS in adult horses and therefore we selected horses used for a wide spectrum of activities, ranging from dressage to racing. Eighty four percent of the horses in the study population had gastric lesions, which is similar to previously reported prevalence data for this geographical region [14]. Unfortunately, there was a limited spectrum of disease in the study population, with a predominance of small single lesions and a noticeable absence of extensive lesions with areas of apparent deep ulceration. As discussed earlier, this has the potential to skew the results by virtue of the fact that permeability of sucrose is directly proportional to the surface area of the damaged gastric mucosa available for permeation. An additional limitation of the study was the fact that that a proportion of the horses in this study (58/101) showed no clinical signs of gastric ulceration at the time of sucrose testing. There is currently little evidence to suggest a direct cause-and-effect relationship between clinical signs of EGUS and the presence, severity or location of gastric ulcers in adult horses [1] and, therefore, it is possible that the diagnostic accuracy of the sucrose test would be improved when testing a population of horses that were all demonstrating clinical signs at the time of gastroscopy.

## Conclusions

Blood sucrose was neither a sensitive nor specific test for detecting EGUS in this population of adult horses with naturally occurring disease. This study included both horses with and without clinical signs of gastric ulceration. Further studies aimed at evaluating the performance characteristics of the test in a selected population of horses demonstrating clinical signs consistent with EGUS may be warranted. Given the limitations of endoscopy, due consideration should also be given to alternative methods for comparison of blood sucrose with a gold standard (Additional file 1).

## Additional file

**Additional file 1.** Estimation of the sensitivity, specificity and predictive values of blood sucrose as a screening test for EGUS in adult horses using Bayesian latent class methods.

## Abbreviations

BW: body weight
EGUS: equine gastric ulcer syndrome
GL: gastric lesion
GDL: glandular lesions
SQL: squamous lesion
CSL: clinically significant lesion
GC-FID: gas chromatography-flame ionization detection
ROC: receiver operator characteristic
AUC: area under the curve
Se: sensitivity
Sp: specificity
PPV: positive predictive value
NPV: negative predictive value
95% CI: 95% confidence intervals
SD: standard deviation

## Footnotes

1 Domosedan, Elanco Animal Health, UK.

2 Butador, Chanelle Vet animal health, UK.

3 Kidesokeri 530, Sucros Oy, Finland.

4 R Foundation for Statistical Computing, Vienna, Austria.
