# Supplementary material for: Diagnostic accuracy of blood sucrose as a screening test for equine gastric ulcer syndrome (EGUS) in adult horses
Source: Acta Vet Scand. 2017 Mar 11;59:15. doi: 10.1186/s13028-017-0284-1 (PMC5346197; doi:10.1186/s13028-017-0284-1)
Supplement: Supplementary file 1 — Additional file 1. Estimation of the sensitivity, specificity and predictive values of blood sucrose as a screening test for EGUS in adult horses using Bayesian latent class methods. [file 13028_2017_284_MOESM1_ESM.doc]

**Diagnostic accuracy of blood sucrose as a screening test for equine gastric ulcer syndrome (EGUS) in adult horses - Bayesian latent class analysis**

**1. Statistical methodology**

Sensitivity, specificity, predictive values, and lesion prevalence were estimated using Bayesian latent class methods. The model is based on the Hui-Walter paradigm [1] but modified for a Bayesian analysis [2]. The model included adjustment for conditional dependence [3] between the two sucrose concentration tests and similar diagnostic test models have been described in more detail elsewhere [4]. The base model was a three test (Sucrose_45, Sucrose_90, endoscopy) and single population model that included adjustment for conditional dependence in sensitivity and specificity estimates for the two sucrose concentration assays. Sucrose concentration results measured at 45 minutes post-administration were dichotomized into positive and negative using 4.61 μmol/L, 5.80 μmol/L, 7.86 μmol/L, and 4.61 μmol/L cutoffs for diagnosis of gastric lesions in general, glandular lesions, squamous lesions, and clinically important lesions, respectively. Evaluated cutoffs were 4.57 μmol/L, 6.05 μmol/L, 8.24 μmol/L, and 5.87 μmol/L for sucrose concentrations measured 90 minutes post-administration for diagnosis of gastric lesions in general, glandular lesions, squamous lesions, and clinically important lesions, respectively. Diffuse, mildly informative, prior probability distributions (Table 1) were elicited based on published literature and expert opinion of the authors. Markov chain Monte Carlo (MCMC) techniques were implemented in available statistical software (WinBUGS Version 1.4, MRC Biostatistics Unit, Cambridge, UK). Iterate values of the MCMC process were expected to be highly correlated and only every 10^th^ iterate was retained to alleviate this concern. Convergence was assessed by evaluating plots of model parameter iterates and by calculating the Gelman-Rubin statistic. The first 200,000 iterations were discarded as the burn-in and inferences were made based on the subsequent 40,000. Median values were used as point estimates and 95% probability intervals (PI) were calculated as the 2.5th to 97.5th percentiles of the posterior distributions.

**2. Results**

**Table 1.** Beta prior probability distributions used in the Bayesian latent class analysis to estimate sensitivity and specificity of test to identify gastric ulcers in adult horses.

| **Population and tests** | **Measure** | **Prior probability distribution (**β**)** | **Mean** | **Median** | **90% probability interval** |
| --- | --- | --- | --- | --- | --- |
| Ulcer | Prevalence | 5,5 | 0.50 | 0.50 | 0.251, 0.749 |
|  |  |  |  |  |  |
| Endoscopy | Sensitivity | 8, 2 | 0.80 | 0.82 | 0.571, 0.959 |
|  | Specificity | 99, 1 | 0.99 | 0.99 | 0.970, 0.999 |
|  |  |  |  |  |  |
| Sucrose 45 | Sensitivity | 5,5 | 0.50 | 0.50 | 0.251, 0.749 |
|  | Specificity | 5,5 | 0.50 | 0.50 | 0.251, 0.749 |
|  |  |  |  |  |  |
| Sucrose 90 | Sensitivity | 5,5 | 0.50 | 0.50 | 0.251, 0.749 |
|  | Specificity | 5,5 | 0.50 | 0.50 | 0.251, 0.749 |

*Uniform (non-informative) prior where all values between 0 and 1 are equally likely.

**Table 2.** Overall prevalence of gastric lesions identified via endoscopy in 101 study horses

|  | **Gold standard*** | **Bayesian LC†** |
| --- | --- | --- |
| **Lesion type** | **Prevalence % (95% CI)** | **Prevalence % (95% PI)** |
| Gastric lesions | 83 (75, 90) | 85 (75, 93) |
| Glandular lesions | 70 (61, 79) | 82 (72, 92) |
| Squamous lesions | 53 (44, 63) | 73 (62, 85) |
| Clinically significant lesions | 58 (49, 68) | 77 (64, 89) |

CI = confidence interval. PI = probability interval; Bayesian analog of the confidence interval

*Calculated relative to direct observation of lesions via endoscopy as the gold standard

†Based on Bayesian latent class analysis

**Table 3.** Sensitivity and specificity of sucrose concentrations using traditional and Bayesian latent class analyses in 101 horses.

|  |  |  | **Traditional*** | **Bayesian LC†** |
| --- | --- | --- | --- | --- |
| **Lesion type** | **Test** | **Parameter** | **Estimate % (95% CI)** | **Estimate % (95% PI)** |
| Gastric lesion | Sucrose 45^a^ | Sensitivity | 67.9 (57.3, 77.2) | 65.3 (54.9, 74.8) |
|  |  | Specificity | 52.9 (29.7, 75.2) | 48.7 (26.8, 69.2) |
|  | Sucrose 90^b^ | Sensitivity | 78.6 (68.8, 86.4) | 78.3 (68.6, 85.8) |
|  |  | Specificity | 47.1 (24.8, 70.3) | 57.3 (36.6, 78.4) |
|  | Endoscopy | Sensitivity | NA | 79.4 (69.4, 88.6) |
|  |  | Specificity | NA | 99.3 (96.1, 1.0) |
|  |  |  |  |  |
| Glandular lesion | Sucrose 45^a^ | Sensitivity | 54.9 (43.3, 66.2) | 52.3 (41.9, 62.5) |
|  |  | Specificity | 56.7 (38.7, 73.4) | 71.2 (46.6, 88.8) |
|  | Sucrose 90^b^ | Sensitivity | 66.2 (54.6, 76.5) | 64.4 (54.2, 73.5) |
|  |  | Specificity | 43.3 (26.6, 61.3) | 31.6 (12.6, 57.3) |
|  | Endoscopy | Sensitivity | NA | 78.9 (67.0, 89.3) |
|  |  | Specificity | NA | 99.3 (96.1, 1.0) |
|  |  |  |  |  |
| Squamous lesion | Sucrose 45^a^ | Sensitivity | 50.0 (36.8, 63.2) | 48.2 (37.3, 59.2) |
|  |  | Specificity | 68.1 (53.8, 80.2) | 54.0 (29.4, 73.1) |
|  | Sucrose 90^b^ | Sensitivity | 57.4 (44.0, 70.0) | 52.2 (41.0, 63.2) |
|  |  | Specificity | 72.3 (58.3, 83.7) | 81.0 (60.9, 93.0) |
|  | Endoscopy | Sensitivity | NA | 76.9 (63.1, 88.3) |
|  |  | Specificity | NA | 99.3 (96.1, 1.0) |
|  |  |  |  |  |
| Clinically significant lesion | Sucrose 45^a^ | Sensitivity | 74.6 (62.4, 84.4) | 68.3 (57.3, 78.1) |
|  |  | Specificity | 50.0 (35.1, 64.9) | 56.3 (34.4, 76.2) |
|  | Sucrose 90^b^ | Sensitivity | 76.3 (64.2, 85.8) | 69.6 (58.6, 79.3) |
|  |  | Specificity | 52.4 (37.4, 67.1) | 61.0 (39.3, 80.4) |
|  | Endoscopy | Sensitivity | NA | 67.9 (56.3, 80.5) |
|  |  | Specificity | NA | 99.3 (96.2, 1.0) |

CI = confidence interval. PI = probability interval; Bayesian analog of the confidence interval. NA = not able to calculate since endoscopy is considered the gold standard reference test.

*Calculated relative to direct observation of lesions via endoscopy as the gold standard

†Based on Bayesian latent class analysis with sucrose tests evaluated at the respective cutoffs^a,b^ and endoscopy assumed to be an imperfect test.

^a^Evaluated cutoffs were 4.61 μmol/L, 5.80 μmol/L, 7.86 μmol/L, and 4.61 μmol/L for sucrose concentrations measured 45 minutes post-administration for diagnosis of gastric lesions in general, glandular lesions, squamous lesions, and clinically significant lesions, respectively.

^b^Evaluated cutoffs were 4.57 μmol/L, 6.05 μmol/L, 8.24 μmol/L, and 5.87 μmol/L for sucrose concentrations measured 90 minutes post-administration for diagnosis of gastric lesions in general, glandular lesions, squamous lesions, and clinically significant lesions, respectively.

**Table 4.** Predictive value positive (PVP) and predictive value negative (PVN) estimated for sucrose concentrations using traditional and Bayesian latent class analyses in 101 horses. Bayesian and traditional calculations were performed for the same sample prevalences determined assuming that endoscopy is a perfect gold standard.

|  |  |  | **Traditional*** | **Bayesian LC†** |
| --- | --- | --- | --- | --- |
| **Lesion type** | **Test** | **Parameter** | **Estimate % (95% CI)** | **Estimate % (95% PI)** |
| Gastric lesion | Sucrose 45^a^ | PPV | 87.7 (78.0, 94.1) | 87.6 (78.0, 94.7) |
|  |  | PPN | 25.0 (12.9, 40.9) | 20.3 (7.2, 37.6) |
|  | Sucrose 90^b^ | PPV | 88.0 (79.1, 94.0) | 91.1 (81.7, 97.0) |
|  |  | PPN | 30.8 (15.4, 50.2) | 32.6 (14.5, 50.9) |
|  |  |  |  |  |
| Glandular lesion | Sucrose 45^a^ | PPV | 75.0 (62.0, 85.3) | 89.5 (77.7, 96.5) |
|  |  | PPN | 34.7 (22.4, 48.7) | 24.8 (9.8, 38.9) |
|  | Sucrose 90^b^ | PPV | 73.4 (61.6, 83.2) | 81.0 (69.0, 92.8) |
|  |  | PPN | 35.1 (21.1, 51.4) | 15.8 (5.6, 32.) |
|  |  |  |  |  |
| Squamous lesion | Sucrose 45^a^ | PPV | 64.3 (49.1, 77.6) | 74.4 (63.1, 86.0) |
|  |  | PPN | 54.2 (41.5, 66.6) | 27.1 (10.4, 45.2) |
|  | Sucrose 90^b^ | PPV | 78.5 (55.8, 82.5) | 88.6 (76.5, 95.9) |
|  |  | PPN | 59.6 (46.6, 71.8) | 37.9 (19.4, 54.1) |
|  |  |  |  |  |
| Clinically significant lesion | Sucrose 45^a^ | PPV | 67.7 (55.6, 78.2) | 83.9 (70.8, 93.7) |
|  |  | PPN | 58.3 (41.9, 73.5) | 35.1 (14.4, 54.9) |
|  | Sucrose 90^b^ | PPV | 69.2 (57.3, 79.5) | 85.7 (72.7, 94.7) |
|  |  | PPN | 61.1 (44.6, 75.9) | 37.9 (16.3, 57.5) |

CI = confidence interval. PI = probability interval; Bayesian analog of the confidence interval. NA = not able to calculate since endoscopy is considered the gold standard reference test.

*Calculated relative to direct observation of lesions via endoscopy as the gold standard

†Based on Bayesian latent class analysis with sucrose tests evaluated at the respective cutoffs^a,b^ and endoscopy assumed to be an imperfect test.

^a^Evaluated cutoffs were 4.61 μmol/L, 5.80 μmol/L, 7.86 μmol/L, and 4.61 μmol/L for sucrose concentrations measured 45 minutes post-administration for diagnosis of gastric lesions in general, glandular lesions, squamous lesions, and clinically significant lesions, respectively.

^b^Evaluated cutoffs were 4.57 μmol/L, 6.05 μmol/L, 8.24 μmol/L, and 5.87 μmol/L for sucrose concentrations measured 90 minutes post-administration for diagnosis of gastric lesions in general, glandular lesions, squamous lesions, and clinically significant lesions, respectively.

3**. References**

[1] Hui SL, Walter SD: 1980, Estimating the error rates of diagnostic tests. Biometrics 36:167-171.

[2] Enøe C, Georgiadis MP, Johnson WO: 2000, Estimation of sensitivity and specificity of diagnostic tests and disease prevalence when the true disease state is unknown. Prev Vet Med 45:61-81.

[3] Vacek PM. The effect of conditional dependence on the evaluation of diagnostic tests. Biometrics 1985;41:959–968.

[4] Fosgate GT, Urdaz-Rodriguez JH, Dunbar MD, Rae DO, Donovan GA, Melendez P, Dobek GL, Alleman AR. Diagnostic accuracy of methods for detecting Anaplasma marginale infection in lactating dairy cattle of Puerto Rico. J Vet Diagn Invest 2010;22:192-199.
